# SELECTIVE NARROWING OF PERIPHERAL SOCIAL NETWORKS PREDICTS POOR LONG-TERM COGNITION IN OLD AGE

**DOI:** 10.1093/geroni/igz038.621

**Published:** 2019-11-08

**Authors:** Hsiao-Wen Liao, Yochai Z Shavit, Laura L Carstensen

**Affiliations:** Stanford University, Stanford, California, United States

## Abstract

Socioemotional selectivity theory posits that emotionally meaningful goals such as spending precious time with close family and friends are prioritized in late life as a function of limited future time horizons. Research documents that older individuals include a smaller proportion of peripheral social partners than younger individuals in their social networks, and that this selectivity is associated with better daily emotional experience (English & Carstensen, 2014). Such limitation of social partners, however, might adversely affect cognitive function in the long run, since exposure to novel and cognitively stimulating environments has been tied to better cognitive functioning (Park et al., 2014). The current study examined the long-term association between proportions of peripheral social partners in older adults’ social networks and cognitive performance. Sixty-one older participants (Mage = 71.53) reported the size of their inner, middle, and outer social circles using the Social Convoy Questionnaire (Kahn & Antonucci, 1980) and completed Digit Span Backward, Digit Span Forward, and Digit Symbol tasks at baseline and five years later. Results of multiple regression analysis show that participants who had a smaller proportion of social partners in their outer social circle at baseline performed poorer on the Backward Span task assessed five years later than those with a larger outer circle proportion. Results hold controlling baseline cognition, physical health, age, SES, education, and trait openness. We discuss the findings in terms of potential tradeoffs between the age-related social selection and working memory in the long run.

